# RF-Powered Low-Energy Sensor Nodes for Predictive Maintenance in Electromagnetically Harsh Industrial Environments

**DOI:** 10.3390/s21020386

**Published:** 2021-01-08

**Authors:** Giacomo Paolini, Marco Guermandi, Diego Masotti, Mazen Shanawani, Francesca Benassi, Luca Benini, Alessandra Costanzo

**Affiliations:** 1 DEI—Department of Electrical, Electronic and Information Engineering “Guglielmo Marconi”, University of Bologna, 40136 Bologna, Italy; marco.guermandi@unibo.it (M.G.); diego.masotti@unibo.it (D.M.); mazen.shanawani@unibo.it (M.S.); francesca.benassi9@unibo.it (F.B.); luca.benini@unibo.it (L.B.); alessandra.costanzo@unibo.it (A.C.); 2 ARCES—Advanced Research Center on Electronic Systems “Ercole De Castro”, University of Bologna, 40125 Bologna, Italy; 3 D-ITET—Department of Information Technology and Electrical Engineering, ETH Zürich, 8092 Zürich, Switzerland; 4 DEI—Department of Electrical, Electronic and Information Engineering “Guglielmo Marconi”, University of Bologna, 47521 Cesena, Italy

**Keywords:** electromagnetically harsh environments, far-field wireless power transfer, industrial internet of things, rectenna, wireless sensor networks

## Abstract

This work describes the design, implementation, and validation of a wireless sensor network for predictive maintenance and remote monitoring in metal-rich, electromagnetically harsh environments. Energy is provided wirelessly at 2.45 GHz employing a system of three co-located active antennas designed with a conformal shape such that it can power, on-demand, sensor nodes located in non-line-of-sight (NLOS) and difficult-to-reach positions. This allows for eliminating the periodic battery replacement of the customized sensor nodes, which are designed to be compact, low-power, and robust. A measurement campaign has been conducted in a real scenario, i.e., the engine compartment of a car, assuming the exploitation of the system in the automotive field. Our work demonstrates that a one radio-frequency (RF) source (illuminator) with a maximum effective isotropic radiated power (EIRP) of 27 dBm is capable of transferring the energy of 4.8 mJ required to fully charge the sensor node in less than 170 s, in the worst case of 112-cm distance between illuminator and node (NLOS). We also show how, in the worst case, the transferred power allows the node to operate every 60 s, where operation includes sampling accelerometer data for 1 s, extracting statistical information, transmitting a 20-byte payload, and receiving a 3-byte acknowledgment using the extremely robust Long Range (LoRa) communication technology. The energy requirement for an active cycle is between 1.45 and 1.65 mJ, while sleep mode current consumption is less than 150 nA, allowing for achieving the targeted battery-free operation with duty cycles as high as 1.7%.

## 1. Introduction

Over the last few years, the industrial internet of things (IIoT) has become a hot research and development topic. The IIoT leverages wireless sensor network (WSN) technologies to enable untethered and seamless connectivity in industrial environments. Several solutions have been tailored to the goal of continuously monitoring key components with untethered sensors to track in real-time rotation, position, speed, temperature, and pressure [1].

One of the key desired characteristics of an IIoT wireless sensor node is the capability to operate autonomously from energy harvesting (EH) rather than relying on bulky batteries, which have a limited lifetime, especially at high temperatures, and might require multiple replacements over the lifetime of a monitored artifact, thereby increasing maintenance cost. Several implementations of such sensors have been presented in the literature, e.g., relying on solar [2,3], wind [3], kinetic [4], thermal [5], thermoelectric [6] or piezoelectric [7] energy harvesting. The main limitations of these solutions are the unreliability of harvesting sources and the need for harvesters to be tailored to the specific deployment scenario.

The development of wireless power transfer (WPT) solutions and the reduction of the node power consumption enabled untethered sensor powering with a reliable and well-controlled energy source, with the two-fold advantage of getting rid of battery cost and environmental impact as well as abating the personnel and material cost related to battery replacements [8,9,10,11]. Predictive maintenance is becoming one of the most promising application targets of these battery-free nodes to enable continuous full-lifetime monitoring of the most critical parts of mission-critical equipment to prevent failure [12].

Companies have placed great emphasis on these goals and are seeking solutions to power the condition-monitoring sensor nodes through radiofrequency (RF) waves. As an example, near-field inductive solutions have been adopted in that sense [13,14,15], as well as prototypes exploiting far-field transmission [16]. RFID technology for quasi-total metallic environments is well developed. For instance, in [17], an antenna tag topology is presented with the aim of being inserted in close contact with metal tools for industrial plants. In addition, many different approaches have been explored towards concepts of wireless information and power transfer (WIPT) [18], and simultaneous wireless information and power transfer (SWIPT) [19], both for consumer and for industrial purposes.

**Figure 1:**
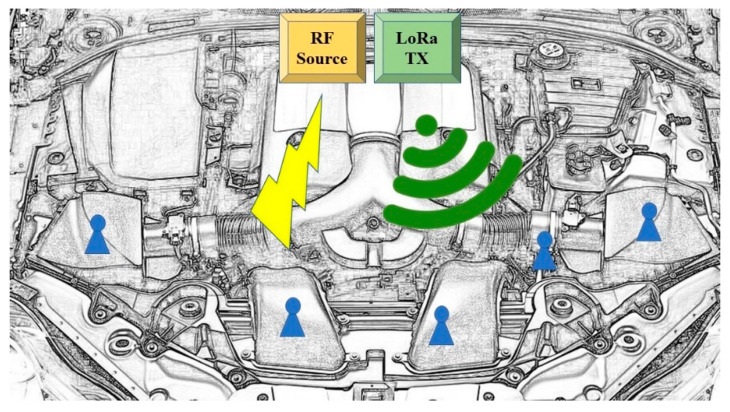
Illustrative example of the envisioned scenario for a WSN system energized by WPT technology in a harsh automotive scenario. The sensor nodes are represented in blue, the power source (yellow), and the communication hub (green) are also shown in one of the possible locations.

Figure 1 represents a sketch of the fully customized WPT and WSN designed system for a specific automotive application example. A major highlight is the possibility to conjugate the electromagnetic far-field WPT capabilities together with the low-power sensor and communication part that is conceived to work in the same frequency band. This allows the simultaneous wireless transfer of power and information, with possibility to store a certain amount of energy enabling the full-cycle activities of a microcontroller unit, a real-time clock, a three-axis accelerometer, but above all bi-directional communication with a Long Range (LoRa) transceiver at 2.4 GHz. 

The main practical outcome of this work for the exemplary automotive application is to demonstrate, for the first time, the possibility to deploy battery-less miniaturized wireless sensor nodes to be placed in correspondence of key points of the car engine, wirelessly powered in accordance with regulations, and monitor temperature and tridimensional acceleration, in order to minimize the need of maintenance for the equipment that is obviously highly time- and money-consuming.

For the 2.45 GHz feeding elements (illuminators), to comply with the highly complex metallic environment where the system operates, a 3D conformal antenna arrangement has been designed, composed of three circularly polarized patch antennas rotated by 45° from each other and incoherently fed by three identical disjointed transmitters.

For the battery-less sensor design, the same operating frequency band has been used for both WPT and LoRa communication and two antennas have been co-located on the sensor node to accomplish these two operations. A proper selection of the electromagnetic (EM) spectrum within the band has been chosen to prevent destructive interference of the high WPT signal on the low-power communication signals. This is demonstrated in this work by a measurement campaign to control the isolation of the two antennas of the nodes and interference tests when RF power and wireless communication are simultaneously running. The node implements several strategies to minimize its energy requirements and guarantee battery-less operation. These strategies are described in the paper and include the use of ultra-low-power communication technology (LoRa), minimization of both sleep and active currents, careful selection of low leakage storage elements, and a novel and extremely energy-efficient management section allowing the full node to operate with an input power down to 30 μW.

Finally, an in-depth test of the overall system is presented and discussed with measurements both in free-space and in the typical working environment where the present work is proposed to be deployed, namely the engine compartment of a hatchback.

## 2. RF Illuminator Design and Characterization

The system wirelessly providing RF energy to the sensor nodes has been designed by integrating off-the-shelf components and by a dedicated design of its conformal radiating part. First, a MAX2750 voltage-controlled oscillator (VCO, Maxim Integrated, San Jose, CA, USA) is used, providing an output power of −3 dBm at 2.45 GHz. This frequency has been selected because it results in being the best compromise between the shrinking and the efficiency of the antennas (for both the illuminators and the sensor nodes), and with the aim of exploiting a maximum EIRP of 27 dBm for an 8-MHz bandwidth signal, this being a regulation requirement for short range devices (SRD).

The VCO can be dynamically tuned in the 2.4–2.5 GHz band to enable frequency diversity between the powering and the communication operations by sweeping the control voltage (V_TUNE_) between 0.8 and 1.4 V; with the aim of generating the signal at 2.45 GHz (selected frequency devoted to WPT), the correct control value is provided by a voltage regulator element (LT6650, Linear Technology Corporation, Milpitas, CA, USA). This allows a suitable frequency spacing between the WPT signal and the LoRa communication signal (chosen working frequency at 2.401 GHz). However, the illuminator’s tuning capability allows its exploitation in the presence of other low-power protocols, such as Bluetooth low energy (BLE), ZigBee [20], or Wi-Fi.

Hence, the key procedure is to set the channel adopted by the communication protocol far enough from the frequency devoted to WPT, in order to guarantee the coexistence and the simultaneity of these two operations: for these reasons, it is crucial to verify the correspondent frequency spacing in the spectrum between the two center frequencies to reduce the percentage packet error rate (PER) to be lower than 0.1%. From experimental studies, it has been determined that the minimum frequency spacing to be adopted between the LoRa communication channel and the wireless power transmission one is 30 MHz.

The VCO output signal is amplified by the SE2598L RF amplifier (gain: 26 dB, Skyworks Solutions, Irvine, CA, USA), whose output directly feeds the antenna incoherently with respect to the other two paths. In this way, a 23 dBm transmitted power is available at each antenna input port. Patch antennas have been chosen for the illuminator, due to their robustness with respect to the surrounding environment and have been designed to be circularly polarized (CP) with a 4-dB gain in the maximum radiation direction. Hence, the total EIRP for each illuminator channel is 27 dBm, compliant with the current regulations.

The chosen substrate for the printed circuit board (PCB) is RO4360G2 (thickness: 0.610 mm, with ε_r_ = 6.4, tan (δ) = 0.0038, Rogers Corporation, Chandler, AZ, USA); it has been selected to obtain reduced microstrip line widths for the connection between the illuminator components and, at the same time, ensure low dielectric losses, with respect to a standard FR-4 substrate (ε_r_ = 4.3, tan (δ) = 0.025).

**Figure 2:**
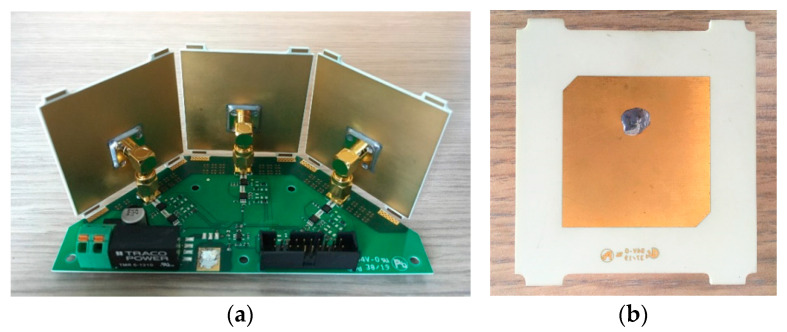
(**a**) Picture of the illuminator feeding circuitry working in the 2.4 GHz band [21] © 2020 IEEE; (**b**) front view of one of the three coaxial-fed circularly polarized patch antennas.

For the patch antenna (Figure 2b), a RO4350B (thickness: 1.524 mm, with ε_r_ = 3.48, tan (δ) = 0.0037, Rogers Corporation, Chandler, AZ, USA) substrate has been used, thicker and less dense with respect to the previous one, to emphasize the radiation performance. The three patch antennas are fed through coaxial cables using a right-angle connector.

Circular polarization has been obtained by means of the corner-trimming technique [22] and chosen to ensure robust WPT performance to the sensors regardless of their orientation with respect to the incident EM waves.

The photo of the entire board of the conformal illuminator is shown in Figure 2a. This compact designed illuminator’s overall dimensions are 13 × 6 × 5.54 cm^3^ and are well suitable to be installed inside internal compartments in industrial machinery and vehicles.

## 3. Design of the Wirelessly Activated Node

In order to face the hostile electromagnetic environment where the battery-less sensor nodes will operate, a dedicated design strategy and a novel layout have been conceived to provide a reliable architecture, whose characteristics must include: (i) compactness, to enable node deployment even on small engine parts; (ii) insensitivity to materials located in close proximity; (iii) energy autonomy, to eliminate batteries and related maintenance; (iv) ultra-low-power sensing and communication operations. To comply with these multiple requirements, a multi-layer architecture has been implemented with two co-located antennas, one for the RF-WPT and one for the communication operations.

**Figure 3:**
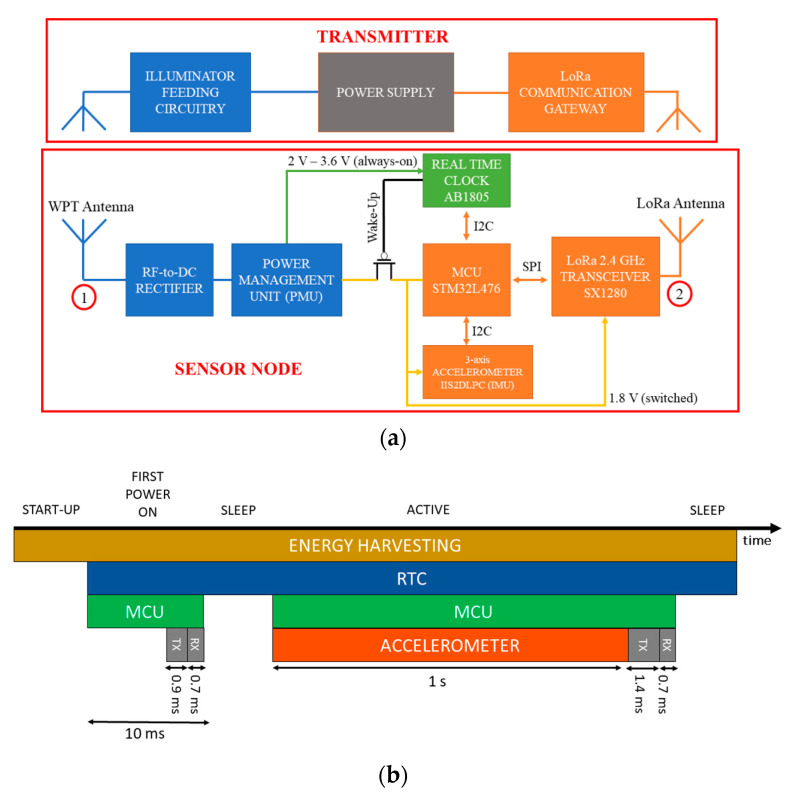
(**a**) Block diagram of the overall system, comprising of the transmitting part (illuminator and LoRa communication gateway), and the wireless sensor node; (**b**) operational modes of the sensor node; colors show which components are active in the different phases.

A simplified diagram of the wireless sensor node is depicted in Figure 3a and consists of the following sub-systems:

- Two co-located antennas, operating in the same frequency band and adopting frequency diverse technique discussed above, one for receiving the energy to be harvested from the WPT, and one for the communication.
- A rectifier for converting the RF signal into a DC signal to be fed to the buck-boost DC/DC converter in the power management section.
- A power management section that efficiently harvests energy at the rectifier’s output to the storage elements and manages the power supply to the sensor node.
- A low power microcontroller unit (MCU, STM32L476RE, STMicroelectronics, Geneva, Switzerland) to control the node peripherals.
- A 2.4 GHz SX1280 transceiver (Semtech Corporation, Camarillo, CA, USA) supporting LoRa communication protocol, feeding a 2450AT42E0100 (Johanson Technology, Camarillo, CA, USA) 2.4 GHz surface mount technology (SMT) above-metal mini chip antenna.
- An ultra-low-power three-axis accelerometer (IIS2DLPC, STMicroelectronics, Geneva, Switzerland) for inertial sensing.
- An ultra-low-power real-time clock (AB1805, Abracon, Spicewood, TX, USA) for duty-cycling the node operation, by turning on the node at specific time intervals only.

It is also noteworthy to observe that using two different antennas for WPT and communication is strategic to minimize energy losses, to avoid RF switches in the EH path. Whereas the performance of the WPT heavily depends on the radiation efficiency of the adopted antenna (which has been therefore custom designed), the communication based on LoRa has been proved to be robust to input signal levels as low as −130 dBm, largely achievable with the compact chip antenna which is integrated on the node.

### 3.1. Node Operations

The selected MCU is a STM32L476RE with 512 kB of flash memory and 128 kB of RAM (random access memory). Of these, approximately 36 kB of flash are used for the code and 16 kB of RAM for the data. This leaves a large amount of flash memory available for either extending code to implement additional on-board processing and/or data logging, without requiring the use of an external memory. In addition, the MCU has been chosen for its exemplary trade-off between power and performance. Clock frequency up to 80 MHz allows fast execution of code to minimize on-time, without sacrificing power consumption (39 μA/MHz). At the same time, it implements several sleep/shutdown modes allowing for reducing power consumption down to 1.1 μA (stop mode) and 30 nA (shutdown mode). Digital (I2C, SPI (serial peripheral interface), UART (universal asynchronous receiver-transmitter)) and analog peripherals (three 12-bits ADCs (analog to digital converters), two 12-bit DACs (digital to analog converters), two PGAs (programmable gain amplifiers), and two ultra-low-power comparators also allow for extending the sensing capabilities of the node to interface to a plethora of analog and digital sensors. The node can operate in the following modes (depicted in Figure 3b):

- START-UP: when power is available at the input, but the storage element is depleted, an undervoltage-lockout (UVLO) circuit keeps the load disconnected from the storage elements until it gets charged up to 2 V. During start-up, only charging operation occurs to minimize losses and charge time and to avoid deadlock conditions, which could happen with an extremely low input power level.
- POWER-ON: when the storage element voltage is above 2 V, the node turns on for the first time and transmits a short package to the gateway of the WSN to signal its presence in the system. The gateway replies with an acknowledgment. The MCU programs the real-time clock (RTC) to wake up the node after the scheduled time. The RTC puts the node in an ultra-low-power sleep mode by cutting off the power supply to the load.
- SLEEP MODE: only the RTC is on, with the node power consumption given by the RTC itself and the PMOS (p-type metal oxide semiconductor) load switch leakage currents. Overall current consumption is below 150 nA. The power management section keeps charging the storage element until it reaches the maximum voltage or 3.3 V.
- ACTIVE MODE: after the programmed sleep time has passed, the RTC awakes the node, and the MCU starts acquisition of accelerometer and temperature data over a 1-s window, processes it (for simplicity, we limit ourselves to minimum, maximum and average on the three-axis in this example), and sends the data to the gateway. The gateway replies with an acknowledgment and, eventually, information on the next wake up time. The node programs the RTC and goes back to sleep mode until the RTC wakes it up again.

The protocol is based on LoRa, a low-power, long-range, single-hop wireless communication technology that has been developed for low-power wide-area networks (LPWAN). Originally, sub-GHz industrial, scientific and medical (ISM) bands were adopted, while, more recently, a LoRa solution operating in the 2.4 GHz ISM band has been introduced.

As proof of concept, we implemented a relatively simple ALOHA communication protocol on a star-topology network. However, more complex ones can be implemented by updating the firmware on the nodes. For this application, given the large disparity between the EH and communication link budgets, the parameters that allow maximum efficiency in terms of energy consumption have been adopted. In particular, Spreading Factor was set to 5, Bandwidth to 1625 kHz, Code Rate to 4/5.

### 3.2. Top Layer Radiative Part for Wireless Energy and Data Transfer

**Figure 4:**
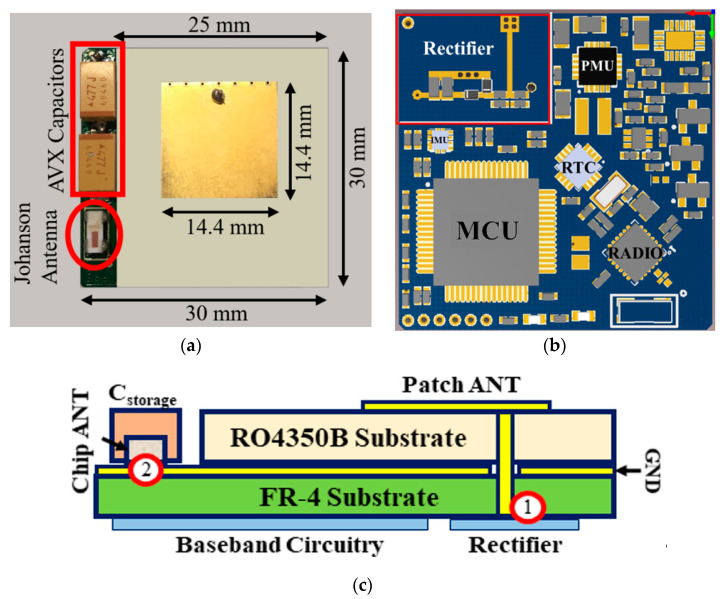
(**a**) Picture of the top layer of the tag, in which it is possible to notice the shorted patch antenna for WPT, the metal-mounted chip antenna, and the storage capacitors; (**b**) bottom layer of the node, with the highlight of the main components and the RF-to-DC rectifier; (**c**) stack-up of the whole sensor node, with the antennas and the capacitors placed on the top layer, the RF and baseband components on the bottom, and the plated through-hole (PTH) connecting patch and rectifier.

With reference to Figure 4c, the multi-layer arrangement with optimized locations of the two antennas has been implemented as follows: the top layer consists of the radiating elements designed to operate in the presence of metal plates; for this purpose, a miniaturized square patch antenna (Figure 4a) has been chosen for WPT. The bottom layer, which is isolated by the top one by means of the ground plane, hosts the RF-to-DC rectifier, the radio, and the baseband subsystems for power managing purposes, except for the two energy storage capacitors that are located on the top layer to reduce the overall node thickness.

We will focus now on the description of the custom patch antenna designed explicitly for the node. The shorting-pin (seven pins, in this case) technique has been adopted to reduce the antenna dimensions (a 55% reduction was obtained), allowing total node dimensions of only 2.50 × 3.00 × 0.32 cm^3^, including substrate and metallization. In particular, two laminates of RO4350B (thickness: 1.524 mm, ε_r_ = 3.48, tan (δ) = 0.0037) have been held together with a prepreg FR-4 film (thickness: 0.120 mm, ε_r_ = 4.5) with the aim of maximizing the realized gain (1.84 dBi), the radiation efficiency (74%), and the bandwidth (35 MHz). It is worth noting that these features have been obtained with an antenna whose dimensions are halved with respect to a traditional coaxial fed patch antenna without shorting pins.

Finally, a plated through-hole has been designed to connect the patch with the rectifier input, located in the node’s bottom layer (Figure 4b).

The mutual position of the two antennas has been designed, with the aid of EM simulations of the whole radiating layer, to establish the maximum patch antenna substrate size reduction, to host the chip antenna, and to minimize the EM coupling and the radiation degradation of both. For these reasons, the chip antenna has been placed at the edge of one of the non-radiative sides of the patch.

In particular, three different antenna configurations have been considered: #1 represents a full 3 × 3 cm^2^ antenna substrate, perfectly overlapping the bottom layer of the node, whereas #2 and #3 correspond to a 0.5-cm cut of the substrate and the ground plane in correspondence with the non-radiative and radiative side edge, respectively. This analysis has been conducted to evaluate how substrate dimensions’ modifications affect the antenna performance in order to create a location in the top layer able to host the two storage capacitors and the SMT chip antenna.

The simulation results show that a significant performance degradation (realized gain: 0.64 dBi) occurs while cutting along the patch antenna radiative side, whereas no significant variations are observed while cutting along the non-radiative one (realized gain: 1.84 dBi). Ultimately, choice #2 (represented in Figure 4a) has been chosen, digging a 3.2 mm-thick inset on the left side of the antenna substrate to host the three above-mentioned components without increasing the overall node thickness.

**Figure 5:**
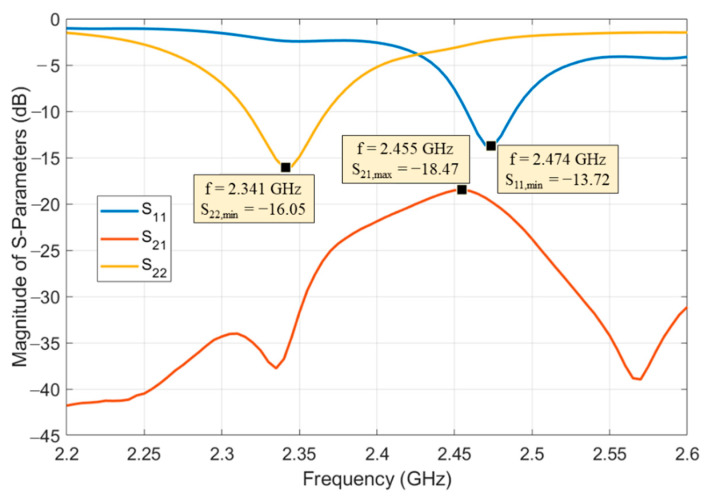
Measured results of the scattering parameters of the two-port antenna on board of the wireless sensor node: ports 1 and 2 are shown in Figure 3a and Figure 4c and correspond to the patch and the metal-mounted chip antennas, respectively.

The resulting mutual decoupling between the two antennas has also been tested by measurement and it is shown in Figure 5, in terms of the transmittance parameter (S_21_) of the resulting two-port antenna: it is possible to notice that an isolation better than 20 dB has been obtained.

### 3.3. Energy Harvesting and Power Management

Given the limited amount of energy that can be harvested (down to few tens of μW), power management needs to be extremely efficient, both in terms of energy harvesting and energy use. As a first remark, the storage elements need to operate with ultra-low leakage currents while storing enough energy to allow node operation. For this reason, we chose to integrate two 470 μF ultra-low-leakage tantalum capacitors (TMJE477K006RCQYA, AVX Corporation, Fountain Inn, SC, USA) on the node, capable of limiting leakage currents to less than 2 μA and coming in a reasonably small form factor (7.3 × 4.4 mm^2^ footprint; thickness: 4.1 mm). With the aim of minimizing the node’s thickness, the capacitors are located on the same side of the PCB allocating the antenna, protruding only 1 mm from the antenna profile.

Regarding the harvesting circuitry, the voltage–doubler rectifier is composed of two Schottky diodes (SMS7630-079LF, Skyworks Solutions, Irvine, CA, USA) and a matching network consisting of one 0.3 pF capacitor and a shorted stub (width: 0.51 mm; length: 5.70 mm). The distributed component choice instead of a lumped one is for losses minimization. The rectifier’s measured performance reveals a 25% of RF-to-DC PCE (power conversion efficiency) [23] with −10 dBm of RF input power, and 34% with −5 dBm.

Figure 4b shows the circuit layout of the power management section. The rectifier output is connected to a buck-boost DC/DC converter (bq25570, Texas Instruments, Dallas, TX, USA) designed explicitly for harvesting energy from low-voltage, low-power sources (down to 100 mV and 15 μW). According to the literature [24], the maximum RF-to-DC efficiency is reached when the rectifier circuit is loaded to ~50% of its open-circuit voltage V_OPEN_, i.e., the voltage at the output of the rectifier when the load is absent (open-circuit condition). For this reason, the bq25570 maximum power point tracker (MPPT) sampling network has been programmed in that sense, allowing for measuring the open-circuit input voltage every 16 s, and then transferring the maximum amount of power currently available to the output.

**Figure 6:**
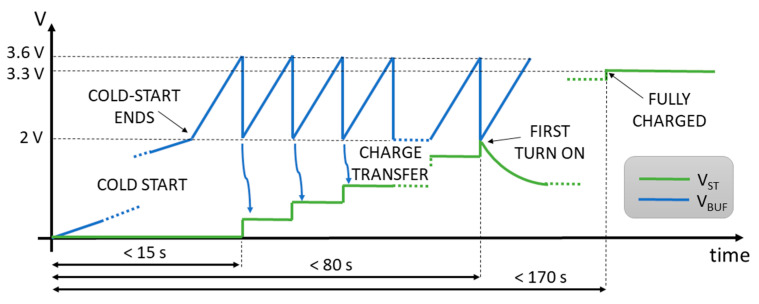
Mechanism to efficiently manage charging of a fully depleted storage capacitance. The reported values are referred to the real case scenario described in Figure 11.

Given the application, the node is expected to be unable to receive any input power when the vehicle is turned off for a long time. Therefore, the capacitors will likely be completely discharged when the vehicle is started. The main issue affecting such a scenario is that the bq25570 operates in a very low-efficiency mode (less than 10%) till the output voltage overcomes the cold-start voltage, which is about 2 V. If the DC/DC converter was required to charge the energy storage capacitors (C_STORE_ = 1 mF) directly up to 2 V, with an input power as low as 30 μW, the charging time would be approximately 22 min, unacceptable for several applications where power might be intermittently removed from the system. In order to overcome this problem, a second DC/DC converter, already integrated inside the bq25570, is exploited, which steps-down the voltage at the output of the first DC/DC converter to 3.3 V and is connected to the tantalum capacitors. The step-up DC/DC converter can thus charge only a 22 μF capacitor (C_BUFFER_, whose voltage V_BUF_ is the blue line in Figure 6), thus reducing dramatically the time required to exit the cold-start state and leading to a turn-on time in the range of few seconds, and up to 30 s for the lowest input power level. When V_BUF_ reaches a programmable threshold set to 3.6 V, the bq25570 sets a pin high, signaling that the voltage is sufficient to turn the buck DC/DC converter on. This pin is directly connected to the enable signal of the buck DC/DC converter, which tries to set its output to 3.3 V. Since the energy on C_BUFFER_ is not enough to charge C_STORE_ up to 3.3 V, the DC/DC will turn on very briefly, transferring charge from C_BUFFER_ to C_STORE_, whose voltage V_ST_ (green curve in Figure 6) will slightly increase, till V_BUF_ reduces to 2 V and the buck DC/DC converter gets turned off. The procedure then starts again without the harvester re-entering cold-start, thus reducing the time required to exit cold-start at the beginning of operation by a factor of 42.

Power supply to the node is then provided through an LTC3106 (Analog Devices, Norwood, MA, USA) buck-boost DC/DC converter whose input is connected to the energy storage capacitors through a low leakage PMOS switch controlled by the RTC circuit. This converter can operate down to 700 mV input voltage, allowing the node to be operational with C_STORE_ voltages between 0.7 and 3.3 V, corresponding to 4.8 mJ of available energy.

**Figure 7:**
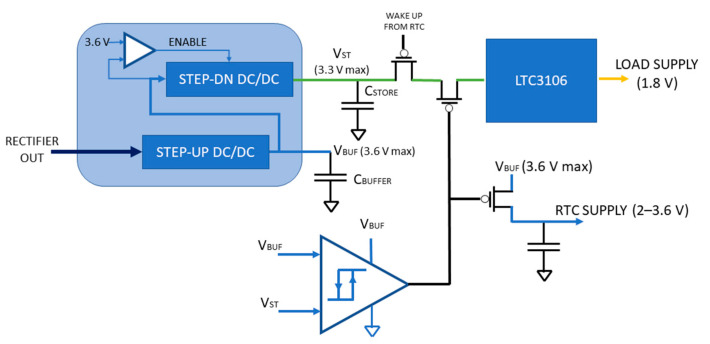
Scheme of the power management section of the wireless sensor node.

A UVLO is also inserted to avoid any load to be connected to the C_BUFFER_ and C_STORE_ at power-up. In fact, RTC current consumption can be in the range of tens of μA when its supply voltage is below the minimum rated supply voltage of 1.5 V. Therefore, for low input power levels, deadlock conditions might occur where the harvester is not capable of overcoming this 1.5 V threshold and exiting the cold-start state. To avoid this occurrence, two additional PMOS load switches are inserted, one at the input of the LTC3106 (in series with the one controlled by the RTC) and one on the RTC power supply. Unfortunately, no fixed voltage is available on the node to determine an absolute threshold, after which RTC can be connected to C_BUFFER_. As shown in Figure 7, a comparison between the voltage levels (V_BUF_ and V_ST_) on C_BUFFER_ and C_STORE_ is performed, and power-on is enabled only when V_ST_ reaches 95% of V_BUF_. At power-up and during cold-start operation, V_ST_ will always be lower than V_BUF_, and the comparator output switches only when V_ST_ reaches approximately 2 V. The ultra-low-power comparator is based on a TLV3691, consuming only 75 nA, featuring an internal UVLO circuit to prevent deadlock conditions. A large hysteresis is inserted to avoid the node from turning off again as soon as C_STORE_ discharges during regular operation.

### 3.4. Sensing, Communication, and Control

When the RTC connects the load switch at the input of the LTC3106, the node gets fully powered and starts its normal operation. At wake up, the SMT32L476 configures the SX1280 transceiver in sleep mode to minimize its power consumption while idle (1 μA typical current consumption). It then configures the IIS2DLPC inertial measurement unit (IMU) for temperature and acceleration data measurement through the I2C interface. The IMU features a 32-level first in first out (FIFO) buffer and a programmable output pin that awakes the MCU to fetch data in bursts when the FIFO is nearly full. MCU’s current consumption is minimized by appropriate usage of direct memory access (DMA) transfers, careful selection of the core operating frequency (reduced to 2 MHz), and use of sleep modes while waiting for the next interrupt from the IMU. The MCU also performs the statistical analysis on the signal during acquisition.

When data is collected, the MCU wakes up the transceiver and sends a 20-byte payload containing the 2-byte node ID, minimum, maximum, and average acceleration on the three axis, and temperature data. The gateway replies with a 3-byte acknowledgment (2-byte node ID and 1-byte acknowledgment).

The MCU then programs the RTC for the subsequent wakeup through the I2C interface, and the RTC itself sends the node to sleep mode till the next wake up.

## 4. System Measurement Campaigns

### 4.1. Sensor Node Consumption

First, the energy consumption of the different blocks of the node for two different sampling frequencies of the IMU data (400 samples per second (SPS) and 1600 SPS) have been characterized. Detailed energy requirements from the node components to operate during the active mode cycle are presented in Table 1.

For an active mode cycle, the node energy requirement is between 1.45 and 1.65 mJ, depending on the accelerometer’s configuration.

In sleep mode, the current drawn from C_BUFFER_ by the RTC is less than 50 nA. Less than 100 nA is similarly drawn from C_STORE_ by the UVLO circuit and to the leakage current on the PMOS switches.

The average efficiency of the LTC3106 has been estimated between 75 and 85%, depending on the input voltage, leading to an overall energy budget of 2.2 mJ. As the maximum energy available on the capacitors is 4.8 mJ, it is large enough to allow two full active cycles.

**Figure 8:**
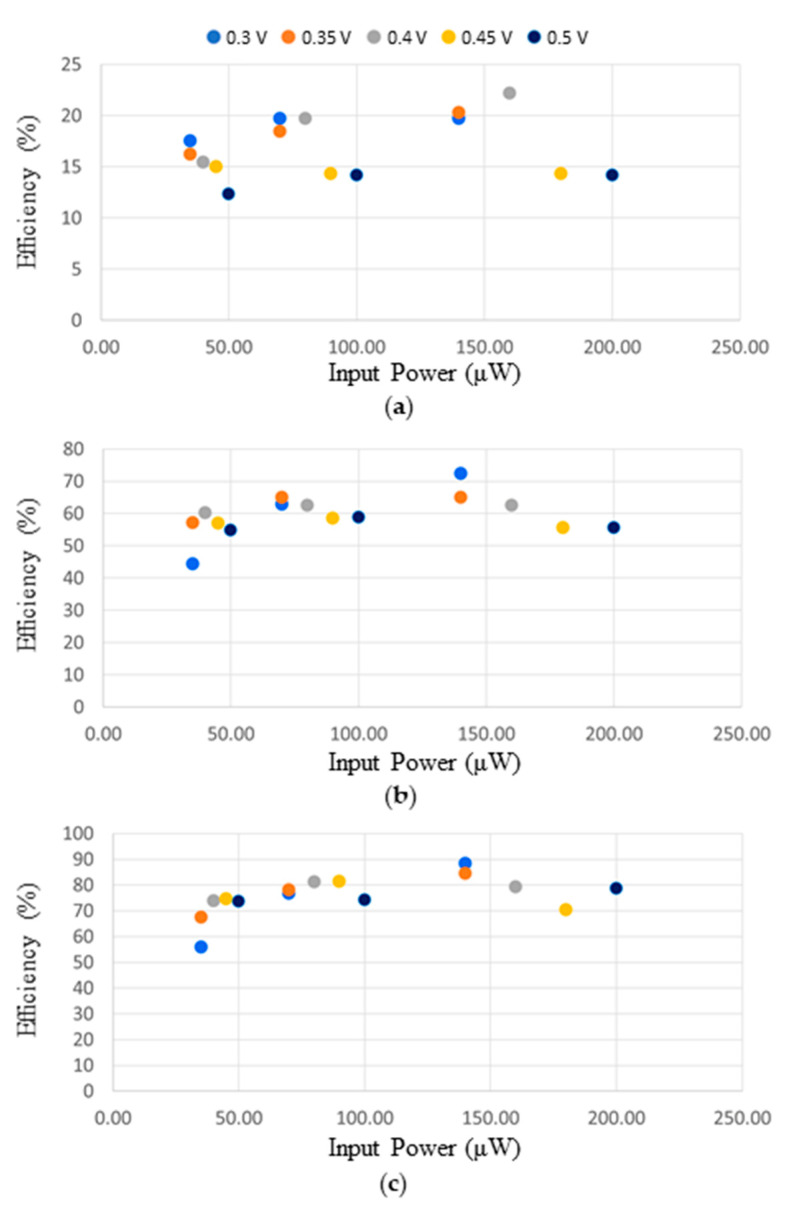
Node’s charging efficiency, versus input power, for different halved open circuit voltages and configurations: (**a**) exit cold-start, (**b**) first power-on, (**c**) fully charged.

To characterize the efficiency with which the storage element gets charged, we connected the input of the bq25570 to a 2450 source meter (Keithley Instruments, Solon, OH, USA). The voltage source was set to the halved open-circuit voltage (ranging from 0.3 to 0.5 V), while the compliance current was set between 100 and 400 μA. For the different configurations, we measured the time required for C_STORE_ capacitance: (i) to start charging, that is exiting the cold-start state and C_BUFFER_ reaching 3.6 V; (ii) to reach 2 V (first power-on); (iii) to reach 3.3 V (fully charged). These measurements are shown in Figure 8. The efficiency is computed as the ratio between the overall energy stored on C_BUFFER_ and C_STORE_ and the power supplied at the input.

As expected, the energy efficiency in charging a capacitor directly connected to the boost converter output (Figure 8a) is very low, between 11 and 22%. However, due to the choice of charging directly only a small C_BUFFER_ and connecting the large storage element C_STORE_ to a second DC/DC converter, the efficiency in fully charging the storage element is improved to values ranging from 52 to 87%.

### 4.2. Rectifying and WPT Characterization

Validation measurements regarding charging times and powers at stake have been performed, adopting a static line-of-sight (LOS) channel on-air, in a typical laboratory environment with the presence of various RF sources, i.e., Bluetooth and WiFi, just to mention two of the signals present and working in the same frequency band of the presented system. These measurements have been carried out for various orientations of the node with respect to the maximum link directions to confirm that the circular polarization of the illuminators transmitting antennas secures the insensitivity of received power to possible orientations of the node antenna.

**Figure 9:**
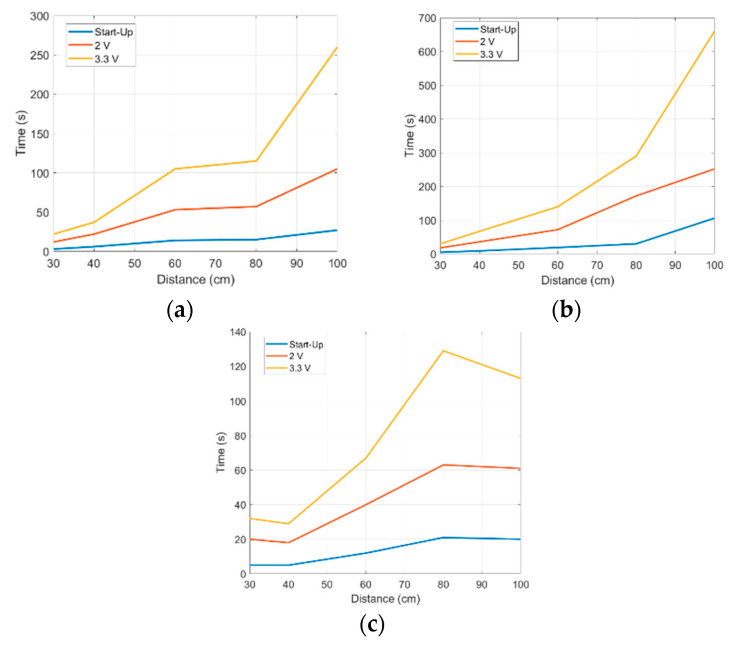
Representation of required times for the nodes to exit the cold-start and charge the storage capacitance at 2 and 3.3 V. Different distances and orientations with respect to the illuminator antennas have been considered: frontal node with (**a**) 0°, (**b**) 30°, and (**c**) 45° inclination with respect to the vertical axis.

In Figure 9, the measurement results obtained during these tests are reported, for increasing illuminator-node distance from 30 up to 100 cm, which is from an almost near-field location up to the far-field condition. The charging times for the PMU cold-start, and for charging the storage capacitance C_STORE_ up to 2 V (first power-on) and up to 3.3 V (complete charge) are shown.

Moreover, it has been noticed that the maximum allowed distance from the illuminator to power the node is less than 120 cm, confirming also that the bq25570 cold-start takes place at 600 mV, at least.

Secondly, another set of measurements has been conducted to characterize the MPPT operations of the bq25570 and verify the accuracy of the nonlinear optimization carried out to maximize the efficiency of the rectifier, in optimum loading conditions, for a reference RF input power (chosen equal to −10 dBm).

The open voltage at the rectifier’s output has been first measured; then, the rectifier DC output has been connected to a potentiometer in order to halve the open voltage (V_OPEN_), which is how the MPPT of the power management unit (PMU) operates. The measured load values registered for V_OPEN_/2 are reported in Table 2. It is worth noticing that, for an illuminator-node distance of 80 cm, the open voltage is 920 mV, which approximately corresponds to an input power of −10 dBm, which matches with a rectifier load (R_LOAD_) of 9.40 kΩ, close to the optimum one predicted by the ADS optimizer (Keysight Technologies, Santa Rosa, CA, USA) (6.44 kΩ) and measured (8.85 kΩ) for an input power of −10 dBm.

### 4.3. Case Study: Measurement Campaign in the Engine Compartment of a Car

In order to validate the overall system in a realistic scenario, a measurement campaign has been carried out in a hatchback engine compartment, which can be considered a practical example of a harsh environment from the EM point of view because it is full of metal parts and obstacles causing multiple reflections of the EM waves.

**Figure 10:**
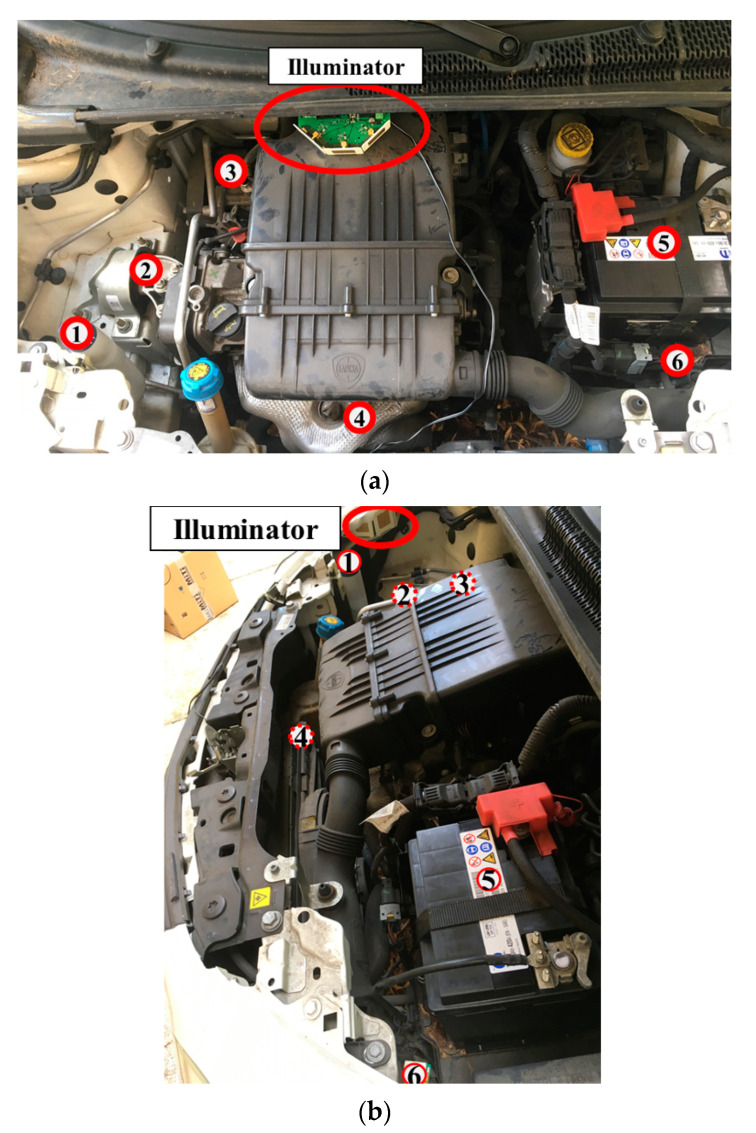
Front and side views of the scenario under test. The RF illuminator and the six positions of the sensors are highlighted both for setup 1 (**a**) and setup 2 (**b**).

The setup consists of several sensor nodes placed in different engine locations and at different distances from the illuminator. The pictures of the two measurement setups are shown in Figure 10 (setup 1: the illuminator is up-frontal; setup 2: the illuminator is down, left-sided): with six nodes distributed all over the engine compartment and only one illuminator present at a time.

## 5. Discussion

Starting from the preliminary design of the RF sources presented in [21], this work describes the full end-to-end design of a wirelessly powered IIoT node designed to guarantee both insensitivity to materials it is placed on, and robustness concerning the ability to store energy. This has been reached with the employment of a conformal illuminator consisting of three circularly-polarized radiating elements, unlike the sensor node power receiving antenna (linearly polarized), with the aim of facing possible changes of the EM waves polarization that can easily happen in metal-rich environments. Furthermore, the choice of a patch antenna at the node side has been done knowing that its bottom layer has to be placed in contact with metallic parts; in this way, its ground plane is virtually enlarged by the metallic components of the engine itself, thus increasing the radiation efficiency.

During the measurement campaign conducted in the engine compartment of the car (see Figure 10), a maximum node-illuminator distance of 112 cm has been considered. It is worth mentioning that sensor nodes are, in this case, placed in a quasi-total metallic environment, which is affected by interfering reflections and non-line-of-sight (NLOS) propagation conditions. Moreover, all the sensors are attached to metallic parts and, in most cases, are also placed close to other metallic elements with the aim of coupling them with the key components of the engine. During these measurements, the bonnet was closed, and the instrumentation was placed outside the car, for the best mimic of realistic operations.

**Figure 11:**
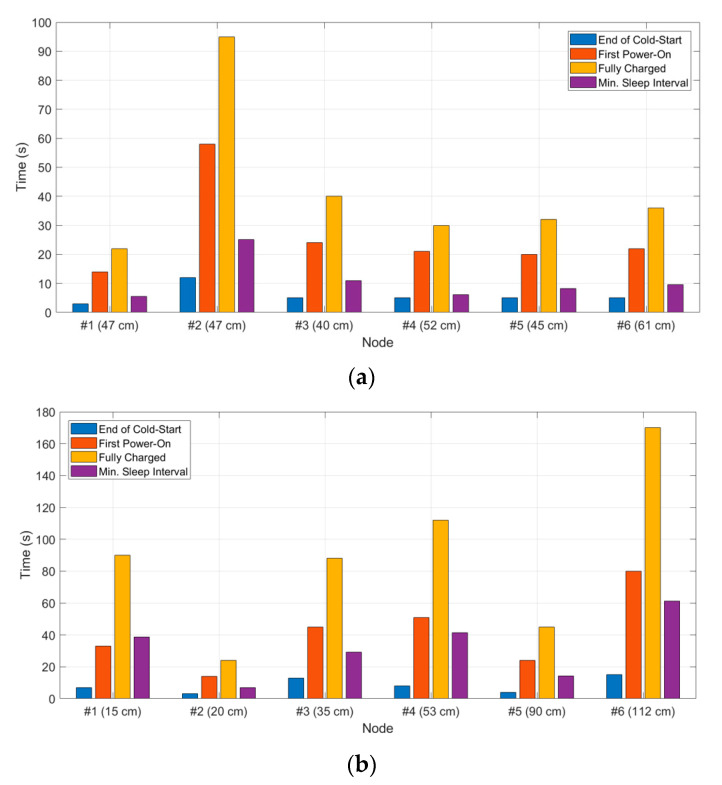
Charging times of the six different nodes for (**a**) setup 1 and (**b**) setup 2.

Figure 11a,b reports a summary of each sensor node’s charging times for the two setups. In particular, the three noteworthy charging times (end of cold-start, first power-on, and full charge) have been monitored, reporting the node-illuminator distance computed along a straight line, together with the minimum sleep time between two subsequent turn-ons of the node (minimum sleep interval).

With regard to the related works, EH techniques for industrial machineries have been investigated in [25] exploiting light, RF, thermal, and kinetic energy. However, at the moment, EM waves coming from RF signals are rarely used for predictive maintenance of mechanical systems. In particular, for what concerns far-field WPT applications [26], useful RF-to-DC PCE are achieved for RF-input power higher than 0 dBm, whereas, in this work, the main goal is to deal with lower power received at the node antenna, given the budget of the radio link at 2.45 GHz and the illuminator-node distances reported in Figure 11. Hence, the targeted low-level of input power in this work is −10 dBm, for which an RF-to-DC PCE of 25% is reached, whereas with −5 dBm a PCE of 34% is achieved: this means that, even with these low input power levels, DC power (25 μW) and voltage (466 mV) at the output of the rectifier (and, equivalently, at the input of the PMU), are sufficient to complete the cold-start phase of the TI bq25570 (taking place from 15 μW and 330 mV).

Finally, circuits for powering wireless sensor networks have been designed making use of commercially available components at the transmitter side in the 915 MHz RFID ultra-high frequency (UHF) band [27], showing a comparable PCE at −10 dBm, but exploiting a receiving tag which integrates a matching network with an area in excess of 16 cm^2^ and a monopole antenna of approximately the same area, which is too large for the specifications and the requirements of our target application. A higher PCE of 45% at −10 dBm is achieved in [28], however operating at 900 MHz, with much larger antenna size of 80 mm × 30 mm × 1.5 mm. In our work, the area of the sensor node, including the antenna, is only 9 cm^2^ (3 × 3 cm^2^) with a thickness of just 4.2 mm, allowing placement in very small cavities, such as inside the engine compartment. It should also be pointed out that neither [27] nor [28] include any sensing on the node.

Regarding the low-power node specifications, the energy requirement for an active cycle results to be between 1.45 and 1.65 mJ, whereas the current consumption is less than 150 nA in sleep mode. This allows for reducing the capacity of the storage elements to only 1 mF, which is critical for reducing its leakage currents and size. The capacity is reduced by a factor 5 with respect to [5], a node which consequently presents a size significantly larger (3 × 5 cm^2^, without accounting for the thermoelectric generator (TEG) and the antenna).

To the authors’ best knowledge, this work represents the first demonstration of a fully self-sustainable, battery-free multi-node sensor system designed and validated for hostile metal-rich scenarios, demonstrating that communication and power transfer to the sensor nodes is robust across different configurations of RF sources and receiving elements. With respect to solutions relying on different energy sources [2,3,4,5,6,7], the energy source itself is integrated and designed as part of the system, guaranteeing full control on the amount of available energy and continuous operation of the wireless sensor nodes.

## 6. Conclusions

In this work, the bottom-up design of a self-deployable wireless RF system for remote monitoring in EM harsh environments has been realized and validated. The system is based on customized energy sources, the illuminators, designed on purpose to be robust in harsh environments and able to sustain the operations of battery-less nodes based on low-power technologies such as LoRa or BLE. The system has been systematically characterized to demonstrate remote node IDs and parameters monitoring communications with no need for batteries on the nodes’ board. The design choices for both the illuminator and the nodes have been carefully tailored with the aim of achieving wireless monitoring in complex environments, with highly efficient WPT capabilities and robustness with respect to the environment involved. The complete operation of the nodes has been demonstrated with charging times compatible with real-life engine monitoring applications.

## Figures and Tables

**Table 1:** Evaluation of required energy for the main components of the node.

Component	Energy
MCU (400 SPS)	380 μJ
MCU (1600 SPS)	510 μJ
IMU (400 SPS)	265 μJ
IMU (1600 SPS)	275 μJ
LoRa Transceiver	405 μJ
Real Time Clock	13.2 μJ
Decoupling Caps (estimate)	70 μJ

**Table 2:** Measurements of the values of the load emulating the bq25570 MPPT operations.

Illuminator–Node Distance	V_OPEN_	R_LOAD_ @ V_OPEN_/2
30 cm	3.24 V	7.58 kΩ
40 cm	2.12 V	10.43 kΩ
60 cm	1.58 V	12.38 kΩ
80 cm	0.92 V	9.40 kΩ

## Data Availability

The data presented in this study are available on request from the corresponding author.
